# Knowledge and Attitudes of Saudi Medical Students about Emergency Management of Traumatic Dental Injuries

**DOI:** 10.3390/ijerph192114249

**Published:** 2022-10-31

**Authors:** Sanaa N. Al-Haj Ali, Ra’fat I. Farah, Serene Alhariqi

**Affiliations:** 1 Department of Orthodontics and Pediatric Dentistry, College of Dentistry, Qassim University, Buraydah 52571, Qassim, Saudi Arabia; 2 Department of Prosthetic Dental Sciences, College of Dentistry, Qassim University, Buraydah 52571, Qassim, Saudi Arabia; 3 College of Dentistry, Qassim University, Buraydah 52571, Qassim, Saudi Arabia

**Keywords:** dental trauma, education, emergency management, medical students

## Abstract

Several studies indicate that physicians lack emergency management knowledge concerning traumatic dental injuries (TDIs), emphasizing the fact that medical students are not taught about this topic. This study aimed to assess the basic knowledge and attitudes of medical students in Saudi Arabia about emergency TDI management. This cross-sectional study recruited a convenience sample of medical students in their clinical years through social networking sites and asked them to answer a pretested internationally accepted questionnaire that included demographic questions, two case scenarios about crown fractures and avulsion of permanent teeth, and self-assessment questions. The data were analyzed statistically using descriptive statistics and the chi-squared test (*p* < 0.05). A total of 761 medical students responded. Only 5.8% of the students reported receiving information about TDIs in their curriculum. Medical students, mainly those ready to graduate, were more knowledgeable of the emergency management of a crown fractured permanent tooth than an avulsed permanent tooth (*p* < 0.0001). However, more than half of the students were unable to differentiate between a primary versus permanent fractured tooth and would manage an avulsed primary or permanent tooth similarly. Regarding students’ attitudes, less than one-quarter of the students (13.5%) were confident about diagnosing TDIs and/or providing emergency management when required (18.1%). Furthermore, only about one-tenth of the students (9.3%) were satisfied with their self-perceived knowledge, and most (71%) reported needing further education about the topic. Medical students in Saudi Arabia have insufficient knowledge about the emergency management of TDIs. Gaps in students’ knowledge of emergency management of avulsion injury were identified in addition to their low confidence level to either diagnose or immediately treat TDIs if required. Students felt dissatisfied with their current knowledge level, this being most prominent among graduating students. There is a need to elaborate the undergraduate medical curriculum in Saudi Arabia to include emergency management of TDIs.

## 1. Introduction

Traumatic dental injuries (TDIs) are considered the most serious oral health problem among children, with violence, sports, road traffic incidents, and falls as the most common causes of such injuries [1]. These injuries can range from crown, root, and/or alveolar bone fracture to luxation and avulsion injuries [2,3]. The dental literature reports a high global prevalence of TDIs with estimates reaching 15.5% to permanent teeth and 23% to primary teeth [4,5]. However, the real estimates might exceed those figures as these injuries are often neglected and could rank fifth if they were included in the list of the world’s most frequent acute/chronic diseases [4,6]. Crown fractures, luxation injuries, and avulsion of permanent teeth require prompt evaluation and treatment to obtain the best possible outcomes as delays in treatment may result in poor prognoses [7]. Diagnosing different TDIs is one of the goals of overall management as the correct diagnosis facilitates optimal treatment and the most favorable outcomes [3].

Physicians have been reported to be the first responders to patients suffering TDIs compared to dentists [8]. Patients who do not have an established dentist [9] or have a dentist, but the injury happened at a time that is outside the working hours of dentists, expect to receive treatment from emergency room physicians at hospitals [8,10]. Consequently, physicians are normally required to manage the emergency treatment phase and stabilize acute TDIs before referring patients to a dentist for continued care [11]. Sadly, studies conducted across the globe have reported low knowledge or confidence level of emergency room physicians and even pediatricians in providing emergency management of TDIs [7,9,12,13,14,15,16,17,18]. One explanation for this was that physicians in emergency departments perhaps do not feel that dental emergencies are the responsibility of the emergency department team [7]. Considering this thinking, Yeng et al. [11,19] called for a change in medical curricula to include more information and skills for the management of TDIs in addition to a better understanding of the importance of early management for medical students. They suggested that education about TDIs should begin with medical students to benefit medical doctors in either general medical practice or specialties that assess and manage trauma. Yet, several studies have reported low knowledge levels of TDIs among medical students [20,21,22], with most of the students (70–90%) reporting not receiving formal training about TDIs in their undergraduate curriculum [20,22,23]. Furthermore, most students perceived knowledge in this regard as not crucial for their future work [22]. 

In Saudi Arabia, the number of pediatric (<14 years old) emergency department visits has been reported to be as high as 3,442,259 in 2021 [24]. Furthermore, a higher prevalence of TDIs (31.4–39%) was reported in some regions of the country than the reported global figures, with crown fractures and avulsion being the most prevalent injuries [25,26,27]. These findings are concerning, especially when they are coupled with a low knowledge level of emergency room physicians in the country about TDIs [12,18]. To the best of our knowledge, no studies to assess the knowledge and attitudes of Saudi medical students about the emergency management of TDIs are available. Ensuring that Saudi medical students are provided with appropriate information about TDIs during their undergraduate studies will eventually be reflected in the way these injuries are immediately managed. Therefore, this study aimed to assess the basic knowledge and attitudes of medical students in Saudi Arabia about the emergency management of TDIs.

## 2. Materials and Methods

### 2.1. Participants

This was a cross-sectional study using a web-based open e-survey conducted on a convenience sample of Saudi medical students who were contacted through the three social networking sites Twitter, Instagram, and WhatsApp. The students were approached through social networking sites due to the COVID-19 pandemic which prohibited direct communication with students from different medical schools given the e-learning educational system which was dominant in the study period (November 2020–April 2021). Furthermore, social media platforms such as Twitter, WhatsApp, Instagram, and others are popular among Saudi health professionals to promote learning in the medical field and disseminate scientific information including research surveys [28]. E-surveys are considered less expensive, easier to disseminate and respond to than paper surveys and allow a larger sample inclusion [29] from a variety of medical schools across the country.

The inclusion criteria for the study were: medical students in clinical years (fourth-sixth year) from any Saudi school (public or private), and those who had an account on the social networking sites Twitter, Instagram, or WhatsApp. Potential participants who met the inclusion criteria were identified by the authors mostly from available official account pages of Saudi medical schools or associations of Saudi medical students and they were contacted via personal social media messages from the accounts of the authors. This method was adopted to guarantee that the targeted population received the survey [30]. There were no preset limits on the number of eligible medical students who should be contacted. Ethical approval for this study was obtained from the Ethical Committee of the College of Dentistry, Qassim University (EA/F-2021-5001). Furthermore, this study was conducted according to CHERRIES guidelines for e-surveys [31].

### 2.2. Study Measures

A structured electronic questionnaire in English was shared with the students during the period from November 2020 through April 2021 (Supplementary Materials). The Google form platform was used because it is available with no requirement for the students to register. We searched the literature to find a valid and reliable questionnaire on knowledge of TDIs among health professionals with particular emphasis on crown fracture and avulsion injuries since these are prevalent in the country [25,26,27]. The most appropriate questionnaire was the one conducted by Raoof et al. [9] among Iranian physicians and dentists. The content validity index of each question was 0.8 to 1, and the internal consistency reliability with Cronbach’s alpha was greater than 0.6. The questionnaire had three parts and aimed to assess several parameters. The first part of the questionnaire assessed the demographic profile of the participants and whether or not they received first-aid training regarding TDIs or had experience with them (four questions). The second part of the questionnaire assessed participants’ knowledge about the emergency management of crown fractures and avulsion injuries through two hypothetical case scenarios, which included nine questions. The third part included self-assessment questions (two) about the participants’ satisfaction with their self-perceived knowledge and interest in further education about the topic. 

In the current study, we slightly modified the questions of the first and third parts of the questionnaire by Raoof et al. [9] to fit the study participants but kept the second part questions unchanged. In the first part, we included questions about the student’s academic level, medical school name and sector, and whether or not the student had received information about emergency management of TDIs in his/her medical curriculum (four questions overall). Furthermore, we added two more questions—to assess students’ confidence levels in terms of diagnosing and treating TDIs among children if required—to the third part of the questionnaire by Raoof et al. [9] (four questions overall). Response categories for the self-assessment questions ranged from strongly disagree to strongly agree. The total number of items in the questionnaire was 17.

A panel of three dental specialists evaluated the final version of the questionnaire in October 2020 before sharing it with the students. Furthermore, the questionnaire was tested among a sample of medical students which included fourth to sixth-year students, and no modifications were made. Pilot testing results were excluded. 

The link for the questionnaire initially included an introduction page to the study where information regarding study aims, participants’ rights, and a consent statement was provided. The students were assured that their responses would remain anonymous and that their participation was voluntary. Furthermore, it was clarified that answering all items of the questionnaire was essential to submit the form and to enable further processing of the responses. Finally, it was clarified that students were allowed to review their answers and change them if required by returning to previous pages and that the sent link allows a single submission to avoid having multiple responses from the same student. The number of pages displayed to the students through the sent link was five. To proceed to the next page of the questionnaire it was mandatory to answer all questions on the previous page. Completing all questions was also necessary to enable form submission. Following the submission of the form, the responses were automatically stored in a secure Excel sheet to which the authors only had access, and at the end of April 2021, the Google form was removed so it was not possible to access the survey link beyond the study period. 

### 2.3. Data Analysis

Statistical analysis of the data was conducted using the SPSS software (version 22.0; IBM Corporation, Armonk, NY, USA (for Windows^®^)). Simple frequency distributions and percentages of the students’ responses to each question were produced and compared according to the academic level and school sector. Multiple response analysis was done for the question which assessed the participants’ knowledge of storage media of avulsed teeth (the last question of the second case scenario) since that question had more than one option to choose from. The frequency of responses to parts two and three questions of the questionnaire was compared using the chi-squared test (univariate approach). Probability values of *p* < 0.05 were considered statistically significant.

## 3. Results

A total of 761 students responded with completed questionnaires. Since answering all questions was mandatory to allow form submission, the completion rate was 100%. The returned questionnaires covered 25 Saudi medical schools out of 28 available schools [32] with the largest contributions being from King Abdulaziz University (28.5%), followed by King Saud Bin Abdulaziz University (12.9%), Jazan University, and Imam Abdulrahman bin Faisal University (7% each). Seven schools were private and 18 were public schools. Of the students, 91% (n = 692) were from public schools compared to 9% (n = 69) from private schools. The highest proportion of the students (43.7%; n = 333) were in their fifth year followed by fourth and sixth-year students (28.2% and 28%; n = 215, and 213; respectively). Only 5.8% (n = 44) of the students received information about the management of TDIs in their curriculum, of whom 81.8% (n = 36) were from public schools.

In the first case, which addressed a broken upper front tooth in a 9-year-old student, slightly less than one-third of the students (27.9%) answered correctly that the tooth was permanent. The great majority of the students (71.6%) chose correctly that they need to advise the patient to save the tooth and refer the patient to a dentist. A statistically significant difference existed in the responses of students from different levels to both questions of this case scenario (*p* < 0.0001) (Table 1), while no significant difference was found according to the medical school sector (*p* > 0.05).

Table 2 shows the students’ responses (according to their level) in the second case scenario which addressed a 12-year-old healthy and conscious boy with an avulsed tooth and some blood in his mouth. Just 6.4% of the students knew that finding the tooth and doing replantation was the correct answer, while the great majority (72.9%) opted to stop the bleeding first. Less than one-third of the students (28.4%) knew that they should immediately replant the tooth, while a greater proportion (37.2%) preferred to attempt replantation within few hours of the injury. A little more than half of the students (55.3%) answered correctly that the tooth should be held from the crown. A statistically significant difference existed in students’ responses from different levels in their way of holding the avulsed tooth (*p* = 0.014).

When the students were asked about the best management option in case the tooth was dirty and had fallen on the ground, slightly more than one-third of them (34.8%) chose correctly that they need to rinse the tooth gently under tap water and do replantation, while one-quarter of them (24.8%) thought that the tooth should be discarded. The great majority of the students (70.2%) opted that they would investigate for tetanus vaccine in this case, which was correct, while about two-thirds of them (63.9%) would also care about the avulsed tooth had it been a primary one, which was an incorrect option, with a statistically significant difference in responses of students from different levels to this question (*p* = 0.047). 

**Figure 1:**
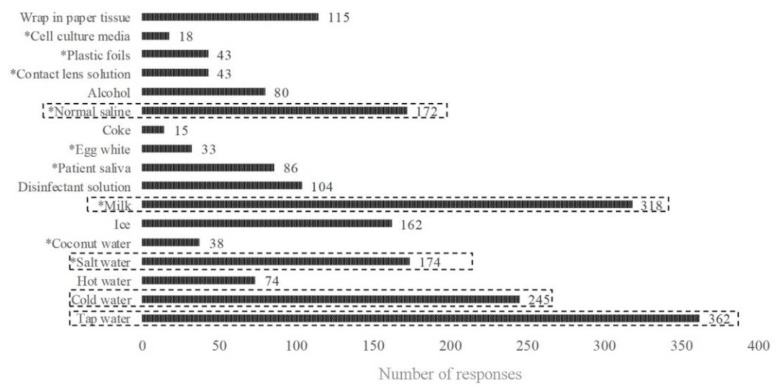
Frequency responses of the students to the question about the storage media of the avulsed tooth. The five most frequently selected media are emphasized. * correct answer.

Figure 1 shows the students’ responses to the question about the storage media of the avulsed tooth. The most frequently reported storage medium was tap water (n = 362) followed by milk (n = 318), cold water (n = 245), salt water, and normal saline (n = 174 and 172, respectively). No significant differences in the students’ responses were found according to the academic level, and overall, no statistically significant differences were found in students’ responses according to the school sector in all questions of the second case scenario (*p* > 0.05) (Table 3).

The students’ attitudes about TDIs including their self-perceived confidence to diagnose as well as provide emergency management of TDIs in children, their satisfaction with their self-perceived knowledge, and their interest in further education about the topic are shown in Table 4 and Table 5. Just 13.5% of the students felt confident to diagnose TDIs in children, and 18.1% of them felt confident to provide emergency management when required. Furthermore, only around one-tenth of the students (9.3%) were satisfied with their self-perceived knowledge. Statistically significant differences existed in students’ responses according to their academic level and school sector with significantly more sixth-year students being insecure about their diagnostic ability and their ability to provide emergency management of TDIs when required as well as being dissatisfied with their self-perceived knowledge level compared to fourth and fifth-year students (*p* = 0.015, 0.033, and 0.002, respectively). Furthermore, significantly more students from public schools were insecure about their diagnostic ability and were dissatisfied with their self-perceived knowledge level compared to students from private schools. The great majority of the students (71%) agreed and strongly agreed that they needed further education about the topic.

## 4. Discussion

The present study is the first to investigate the knowledge and attitudes of Saudi medical students toward the emergency management of TDIs. A few studies have investigated this topic elsewhere using different questionnaires with particular emphasis on avulsion injuries among small samples of medical students, students from a single medical school, medical students from all academic years, or only first-year students [20,21,22,23,33]. According to Ravn, the comparison of studies related to dental trauma is difficult as few epidemiological surveys are comparable due to differences in methodologies and populations [34]. Particular emphasis was given in the present study to exclusively assess the knowledge of a larger sample of medical students in their clinical years as those were the ones closest to graduation and had certainly finished most of their curriculum, including any emergency medicine courses or dental trauma-related courses. Furthermore, these students are exposed to patients during their medical rotations, unlike students from non-clinical years. A comparison of students’ knowledge between students from different clinical years was essential, as compared to students from lower levels, graduating (final year) students should be more knowledgeable because they had a better solid foundation of pre-clinical and clinical medical sciences compared to students from lower clinical levels [19].

The findings of the present study support previous results [20,21,22] that medical students still lack the knowledge of how to manage TDIs. Several gaps in medical students’ knowledge about the emergency management of TDIs were identified, which were more prominent in the case of avulsed permanent teeth. In the case of the fractured tooth, students’ knowledge of tooth identification (primary versus permanent) was deficient, regardless of their academic level. Raoof et al. [9] reported the same finding among Iranian physicians. According to the International Association of Dental Traumatology (IADT) [35,36], the tooth fragment in primary tooth fracture is expelled from the mouth in most cases, so the medical doctor needs only to confirm this and exclude that the fragment has not been ingested, aspirated, or embedded in soft tissues; while for permanent teeth, a crown fracture at the horizontal middle line leaves the possibility of retaining an appreciable piece of the crown, which when retrieved in an intact form, can be easily reattached by the dentist. Luckily, the students, especially those about to graduate, had sufficient knowledge of the immediate emergency action of the fractured permanent tooth, a finding that perhaps reflects students’ exposure to this information from sources that need to be explored in a future study.

In the case of an avulsed permanent tooth, a deficiency in students’ knowledge was evident in the areas which concerned their immediate emergency management of the tooth, the urgency of the replantation, their management of an avulsed primary tooth or a dirty avulsed permanent tooth, and the best storage media for avulsed permanent teeth (five out of seven questions). In those areas, the correct answer was given by less than 50% of the students. Less than one-tenth of the students (6.4%) opted to search for the avulsed tooth and attempt replantation. Moreover, the majority did not know that replantation needed to be immediate (71.6%). Qazi and Nasir [33] reported a similar percentage of Pakistani medical students choosing immediate replantation (5.2%); however, the students in that study were only first-year.

The rest of the studies that concerned medical students reported better student knowledge about the need to replant avulsed permanent teeth. In the study of Ivkošić et al. [22] in Croatia, 46.7% of 135 medical students knew that a permanent tooth can be replanted after avulsion, while the study of Rodrigues et al. [21] in Brazil reported that 20.3% of 305 medical students chose to replant the tooth. Jokic et al. [23] reported the highest percentage of medical students choosing this management option (77.6% of 86 students). In the present study, the students preferred to stop the bleeding first (72.9%). This finding has been previously noted as medical doctors tend to prioritize wounds, bleeding, and bone injuries over tooth injuries [9,20,33], perhaps due to the emergency life support course that they take at the undergraduate level which provides training in how to deal with life-threatening conditions, including bleeding in trauma patients [9]. However, immediate replantation of the avulsed permanent tooth should always precede this, but the doctor must first confirm that the tooth is permanent and that it is clean. In the present study, more than two-thirds of the students reported that they would choose a similar method to care about the avulsed tooth whether primary or permanent, which was incorrect. Primary teeth should not be replanted to avoid potentially causing damage to the permanent tooth, to its eruption, or a medical emergency resulting from aspiration of the tooth [35,37]. If the tooth is dirty, it should be gently rinsed with water, milk, saline, or saliva before replantation [37]. Just about one-third of the students opted to rinse the tooth gently with water if it was dirty while surprisingly one-fourth of them (24.8%) opted to discard the tooth in this situation. 

When immediate replantation of the avulsed tooth cannot be done, storage media should be used to avoid dehydration of the root surface [37]. A noted deficiency of knowledge of the best storage media is evident as students selected tap water followed by milk, and cold water as the most suitable storage media, while the IADT recommends milk followed by Hanks balanced salt solution (HBSS) or saline the most. HBSS was categorized among the cell culture media in the present study; surprisingly, just 18 students chose this medium and still preferred water, which has been repeatedly reported as a poor medium [37], over HBSS and milk. Tap water may induce rapid cell lysis due to its hypotonic properties [38].

Effective education requires the educator not only to identify gaps in knowledge but also to identify attitudes to design an effective strategy to deliver the education [18]. In our study, the great majority of the students (>70%), particularly sixth-year students and those from public schools, reported low confidence levels in terms of diagnosing TDIs in children, were dissatisfied with their self-perceived knowledge, and sought further education about the topic. Sixth-year students also reported low confidence levels in terms of treating TDIs if required. This finding perhaps reflects a lack of education about TDIs in the medical curriculum, which is reflected in knowledge gaps on how to manage avulsion injury in particular. In the present study, the proportion of students who reported receiving information about the topic in their curriculum was almost negligible (5.8%).

In the medical field, the management of traumatic injuries falls within the scope of emergency medicine. Saudi Arabia, despite its wealth and large size, has a relatively new emergency medicine system [39]. Al-Rabaiah et al. [40] previously reported that around 69% of Saudi medical students did not have emergency medicine as an independent module, and 55% of the students did not have it as an elective course. The schools that provided emergency medicine courses in the country were the smaller ones, presumably private, and these courses comprised mostly basic and advanced cardiac life support. These findings perhaps explain the lower confidence level of students from public schools as students of private schools had better chances of receiving a course in emergency medicine. However, the knowledge of medical students from private schools about TDIs did not differ significantly from those of the public schools, which may indicate that emergency medicine courses failed to provide relevant skills and knowledge of TDIs and indicate the need to further elaborate the basic life support courses to include management of TDIs. Alternatively, adding a separate course about dental trauma, perhaps online, could lead to improved student knowledge about such subjects. Recently, Yeng et al. [41] incorporated a dental trauma course for medical students in Australia and found that students perceived the online course to be easily understood for self-learning this topic.

Another suggestion would be improving the access of medical students to simulation practice. In Saudi Arabia, Khattab et al. [39] have reported that simulation-based learning has begun to be utilized by some academic hospitals with institutional capabilities and resources for simulation education. While the availability of this option is limited, according to Aalam et al. [42], this step certainly would help medical students who would less likely see patients with TDIs during their regular clerkships and have simulation facilities in their schools. Zafar et al. [43] recently reported that emergency management of avulsion injury can be easily learned by a majority of dental students (81%) in simulation laboratories with students feeling better prepared to treat patients who present with TDIs in the future following this experience when compared to relying solely on didactic training. Medical students should also be encouraged to engage in webinars, which are often offered free of charge, by experienced dentists or faculty members.

The present study has a few points of strength in addition to limitations that should be addressed. Targeting a larger sample size of students compared to previous studies on medical students from a variety of medical schools including those in rural regions is considered a point of strength. Following a convenience sampling approach which may limit the generalizability of the results, especially when the low number of students from private schools compared to public schools is considered, constitutes a limitation. Furthermore, due to the low number of students who reported receiving information about the topic in their curriculum, investigating the association between having received information and students’ knowledge of the case scenarios was difficult to perform. Furthermore, the case scenarios presented dealt with only two types of TDIs, namely, avulsion and crown fractures, as these are prevalent in the country; however, it is also important that medical students have proper knowledge of the emergency management of other TDIs, such as luxations. Another limitation is the cross-sectional nature of the study, meaning that we cannot infer causation from any of the associations we observed. Furthermore, sending e-questionnaires through social networking sites has some disadvantages, such as recruiting and limiting the responses to the students who have accounts on those sites or are internet active [44], which can result in a skewed distribution of responses received from students of different medical schools. Furthermore, students who have a slow internet connection may not participate since it will likely take a long time for them to access the questionnaire [29]. Nevertheless, it is worth mentioning that while studies that have investigated the knowledge of physicians about TDIs are plenty, those that addressed the knowledge of medical students are extremely limited. Consequently, the baseline data obtained in the present study are still essential for future studies.

## 5. Conclusions

Medical students in Saudi Arabia, regardless of their academic level, have insufficient knowledge about the emergency management of traumatic dental injuries. Several gaps in the knowledge of emergency management of avulsion injury, in particular, were identified. In addition, students were found to have a low confidence level to either diagnose or immediately treat TDIs if required, which was most prominent among graduating students. There is a need to elaborate the undergraduate medical curriculum in Saudi Arabia to include emergency management of TDIs to enhance the students’ theoretical and clinical knowledge.

## Figures and Tables

**Table 1:** The frequency and percentage (N%) of medical students’ responses to the first case scenario according to the academic level and school sector.

Q1 and 2	Tooth Type			Immediate Management		
	Deciduous	Permanent †	Do Not Know	Referral to a Dentist without Keeping the Tooth Fragment	Advise Patient to Save Tooth Fragment and Refer to a Dentist †	Suggest Tooth Extraction
Fourth-year	80 (37.2)	58 (26.9)	77 (35.8)	41 (19.0)	152 (70.6)	22 (10.2)
Fifth year	103 (30.9)	70 (21.0)	160 (48.0)	79 (23.7)	218 (65.4)	36 (10.8)
Sixth year	53 (24.8)	85 (39.9)	75 (35.2)	18 (8.4)	175 (82.1)	20 (9.3)
*p* value	<0.0001 *			<0.0001 *		
Total	236 (31.0)	213 (27.9)	312 (40.9)	138 (18.1)	545 (71.6)	78 (10.2)
Public school	211 (30.5)	190 (27.4)	291 (42)	127 (18.3)	494 (71.3)	71 (10.2)
Private school	25 (36.2)	23 (33.3)	21 (30.4)	11 (15.9)	51 (73.9)	7 (10.1)
*p* value	0.173			0.879		

* statistically significant. † correct answer.

**Table 2:** The frequency and percentage, N(%), of medical students’ responses to the first six questions of the second case scenario according to the academic level.

Q1	Immediate Management							
	Stop the bleeding by applying gentle pressure with a cloth over the injury and advise the patient to rest		Stop the bleeding and then search for the tooth	Look for the tooth and put it back in its socket †		Place the tooth in a handkerchief and refer the child to a dentist		Because of the hopeless prognosis, there is no need to replant the tooth
Fourth-year	100 (46.5)		56 (26.0)	15 (7)		37 (17.2)		7 (3.2)
Fifth year	152 (45.6)		98 (29.4)	16 (4.8)		60 (18.0)		7 (2.1)
Sixth year	85 (39.9)		64 (30.0)	18 (8.5)		40 (18.8)		6 (2.8)
*p* value	0.682							
Total	337 (44.3)		218 (28.6)	49 (6.4)		137 (18.0)		20 (2.6)
**Q2–4**	**Need to investigate for tetanus vaccine**		**The urgency to replant the avulsed tooth**				**Care in case a primary tooth got avulsed**	
	**Yes †**	**No**	**Immediately †**	**Within a few hours**	**Within the same day**	**This is not a crucial factor**	**Yes**	**No †**
Fourth-year	141 (65.6)	74 (34.4)	59 (27.4)	82 (38.1)	44 (20.4)	30 (14)	152 (70.7)	63 (29.3)
Fifth year	237 (71.1)	96 (28.8)	93 (27.9)	114 (34.2)	54 (16.2)	72 (21.6)	205 (61.6)	128 (38.4)
Sixth year	156 (73.2)	57 (26.7)	64 (30)	87 (40.8)	31 (14.5)	31(14.5)	129 (60.5)	84 (39.4)
*p* value	0.194		0.119			0.047 *		
Total	534 (70.2)	227 (29.8)	216 (28.4)	283 (37.2)	129 (17.0)	133 (17.5)	486 (63.9)	275 (36.1)
**Q5 and 6**	**Management in case the tooth was dirty and had fallen on the ground**					**Way of holding the tooth**		
	**Rub away the dirt with a paper tissue and put it back into its socket**		**Clean the tooth with a toothbrush under tap water and put it back into its socket**	**Rinse the tooth gently under tap water and put it back into its socket†**	**Discard the tooth**	**By the crown†**	**By the root**	**Not important (crown or root)**
Fourth-year	20 (9.3)		70 (32.5)	74 (34.4)	51 (23.7)	104 (48.3)	43 (20.0)	68 (31.6)
Fifth year	30 (9.0)		109 (32.7)	99 (29.7)	95 (28.5)	181 (54.3)	51 (15.3)	101 (30.3)
Sixth year	15 (7)		63 (29.6)	92 (43.1)	43 (20.1)	136 (63.8)	33 (15.4)	44 (20.6)
*p* value	0.066					0.014 *		
Total	65 (8.5)		242 (31.8)	265 (34.8)	189 (24.8)	421 (55.3)	127 (16.7)	213 (28)

* statistically significant, † correct answer.

**Table 3:** The frequency and percentage, N(%) of medical students’ responses to the first six questions of the second case scenario according to the school sector.

Q1	Immediate Management							
	Stop the Bleeding by Applying Gentle Pressure with A Cloth over the Injury and Advise the Patient to Rest		Stop the Bleeding and Then Search for the Tooth	Look for the Tooth and Put It Back in Its Socket †	Place the Tooth in a Handkerchief and Refer the Child to A Dentist		Because of the hopeless prognosis, there is no need to replant the tooth	
Public school	311 (44.9)		193 (27.9)	44 (6.3)	125 (18)		19 (2.7)	
Private school	26 (37.6)		25 (36.2)	5 (7.2)	12 (17.3)		1 (1.4)	
*p* value	0.598							
**Q2–4**	**Need to investigate for tetanus vaccine**		**The urgency to replant the avulsed tooth**				**Care in case a primary tooth got avulsed**	
	**Yes †**	**No**	**Immediately †**	**Within a few hours**	**Within the same day**	**This is not a crucial factor**	**Yes**	**No †**
Public school	489 (70.6)	203 (29.3)	194 (28)	259 (37.4)	115 (16.6)	124 (17.9)	443 (64)	249 (35.9)
Private school	45 (65.2)	24 (34.7)	22 (31.8)	24 (34.7)	14 (20.2)	9 (13)	43 (62.3)	26 (37.7)
*p* value	0.209		0.615				0.437	
**Q5 and 6**	**Management in case the tooth was dirty and had fallen on the ground**					**Way of holding the tooth**		
	**Rub away the dirt with a paper tissue and put it back into its socket**		**Clean the tooth with a toothbrush under tap water and put it back into its socket**	**Rinse the tooth gently under tap water and put it back into its socket †**	**Discard the tooth**	**By the crown †**	**By the root**	**Not important (crown or root)**
Public school	56 (8.1)		222 (32)	237 (34.2)	177 (25.6)	391 (56.5)	113 (16.3)	188 (27.1)
Private school	9 (13)		20 (28.9)	28 (40.5)	12 (17.4)	30 (43.5)	14 (20.2)	25 (36.2)
*p* value	0.221					0.113		

† correct answer.

**Table 4:** The students’ attitudes about emergency management of traumatic dental injuries according to school sector (N%).

	Answer Option	Public School	Private School	Total	*p* Value
Confidence in diagnosing different TDIs affecting children	Disagree to strongly disagree	407 (58.8)	28 (40.6)	(435) 57.1	0.03 *
	Neither agree nor disagree	194 (28.0)	29 (42.0)	223 (29.3)	
	Agree to strongly agree	91 (13.1)	12 (17.4)	(103) 13.5	
Confidence in the ability to provide emergency treatment for TDIs	Disagree to strongly disagree	406 (58.6)	29 (42)	435 (57.1)	0.079
	Neither agree nor disagree	167 (24.1)	21 (30.4)	188 (24.7)	
	Agree to strongly agree	119 (17.1)	19 (27.5)	138 (18.1)	
Satisfaction about knowledge level regarding TDIs and their management	Disagree to strongly disagree	485 (70.0)	33 (47.8)	518 (68.1)	0.009 *
	Neither agree nor disagree	147 (21.2)	25 (36.2)	172 (22.6)	
	Agree to strongly agree	60 (8.6)	11 (15.9)	71 (9.3)	
Need further education regarding TDIs and their management	Disagree to strongly disagree	79 (11.4)	5 (7.2)	84 (11)	0.769
	Neither agree nor disagree	121 (17.4)	16 (23.1)	137 (18)	
	Agree to strongly agree	492 (71.0)	48 (69.5)	540 (71)	

TDIs: traumatic dental injuries. * statistically significant.

**Table 5:** The students’ attitudes about emergency management of traumatic dental injuries according to the academic level (N%).

	Answer Option	Fourth-Year	Fifth Year	Sixth Year	Total	*p* Value
Confidence in diagnosing different TDIs affecting children	Disagree to strongly disagree	101 (47.0)	202 (60.6)	132 (61.9)	435 (57.1)	0.015 *
	Neither agree nor disagree	80 (37.2)	89 (26.7)	54 (25.3)	223 (29.3)	
	Agree to strongly agree	34 (15.8)	42 (12.6)	27 (12.6)	103 (13.5)	
Confidence in the ability to provide emergency treatment for TDIs	Disagree to strongly disagree	109 (50.6)	190 (57.1)	136 (63.8)	435 (57.1)	0.033 *
	Neither agree nor disagree	55 (25.5)	84 (25.2)	49 (23.0)	188 (24.7)	
	Agree to strongly agree	51 (23.7)	59 (17.7)	28 (13.1)	138 (18.1)	
Satisfaction about knowledge level regarding TDIs and their management	Disagree to strongly disagree	131 (60.9)	232 (69.6)	155 (72.7)	518 (68.0)	0.002 *
	Neither agree nor disagree	51 (23.7)	74 (22.2)	47 (22.1)	172 (22.6)	
	Agree to strongly agree	33 (15.3)	27 (8.1)	11 (5.1)	71 (9.3)	
Need further education regarding TDIs and their management	Disagree to strongly disagree	24 (11.1)	36 (10.8)	24 (11.2)	84 (11.0)	0.861
	Neither agree nor disagree	44 (20.4)	58 (17.4)	35 (16.4)	137 (18.0)	
	Agree to strongly agree	147 (68.3)	239 (71.7)	154 (72.3)	540 (71.0)	

TDIs: traumatic dental injuries. * statistically significant.

## Data Availability

The datasets used and/or analyzed during the current study are available from the corresponding author upon reasonable request.

## Supplementary Materials

The following supporting information can be downloaded at: https://www.mdpi.com/article/10.3390/ijerph192114249/s1, The research questionnaire used in the study.

## References

[1] Afrashtehfar K.I., Chung J. (2017). Mouthguard use may reduce dentofacial injuries in field hockey players. Evid. Based Dent..

[2] Al-Haj Ali S.N., Algarawi S.A., Alrubaian A.M., Alasqah A.I. (2020). Knowledge of General Dental Practitioners and Specialists about Emergency Management of Traumatic Dental Injuries in Qassim, Saudi Arabia. Int. J. Pediatr..

[3] Srivastava A., Gupta N., Marleau A., Afrashtehfar K.I. (2014). How do I manage a patient with intrusion of a permanent incisor?. J. Can. Dent. Assoc..

[4] Andreasen J.O., Andreasen F.M., Andersson L. (2019). Textbook and Color Atlas of Traumatic Injuries to the Teeth.

[5] Afrashtehfar K. (2022). The rise of e-scooter-related dentofacial trauma calls for immediate safety policymaking. Br. Dent. J..

[6] Petti S., Glendor U., Andersson L. (2018). World traumatic dental injury prevalence and incidence, a meta-analysis-One billion living people have had traumatic dental injuries. Dent. Traumatol..

[7] Yigit Y., Helvacioglu-Yigit D., Kan B., Ilgen C., Yilmaz S. (2019). Dentofacial traumatic injuries: A survey of knowledge and attitudes among emergency medicine physicians in Turkey. Dent. Traumatol..

[8] Liew V.P., Daly C.G. (1986). Anterior dental trauma treated after-hours in Newcastle, Australia. Community Dent. Oral. Epidemiol..

[9] Raoof M., Vakilian A., Kakoei S., Manochehrifar H., Mohammadalizadeh S. (2013). Should medical students be educated about dental trauma emergency management? A study of physicians and dentists in Kerman Province, Iran. J. Dent. Educ..

[10] Martin I.G., Daly C.G., Liew V.P. (1990). After-hours treatment of anterior dental trauma in Newcastle and western Sydney: A four-year study. Aust. Dent. J..

[11] Yeng T., O’Sullivan A.J., Shulruf B. (2020). Medical doctors’ knowledge of dental trauma management: A review. Dent. Traumatol..

[12] Bahammam L.A. (2018). Knowledge and attitude of emergency physician about the emergency management of tooth avulsion. BMC Oral. Health.

[13] Tadin A., Delic D., Delic Jukic I.K., Gorseta K., Gavic L. (2021). Pediatricians’ Knowledge of Emergency Management of Dental Injuries and Use of Mouthguards: A Cross-Sectional Survey. Dent. J..

[14] Needleman H.L., Stucenski K., Forbes P.W., Chen Q., Stack A.M. (2013). Massachusetts emergency departments’ resources and physicians’ knowledge of management of traumatic dental injuries. Dent. Traumatol..

[15] Trivedy C., Kodate N., Ross A., Al-Rawi H., Jaiganesh T., Harris T., Anderson J.E. (2012). The attitudes and awareness of emergency department (ED) physicians towards the management of common dentofacial emergencies. Dent. Traumatol..

[16] Ulusoy A.T., Onder H., Cetin B., Kaya S. (2012). Knowledge of medical hospital emergency physicians about the first-aid management of traumatic tooth avulsion. Int. J. Paediatr. Dent..

[17] Abu-Dawoud M., Al-Enezi B., Andersson L. (2007). Knowledge of emergency management of avulsed teeth among young physicians and dentists. Dent. Traumatol..

[18] Pani S.C., Eskandrani R.M., Al-Kadhi K., Al-Hazmi A. (2015). Knowledge and attitude toward dental trauma first aid among a sample of emergency room personnel across Saudi Arabia. Saudi J. Oral. Sci..

[19] Yeng T., O’Sullivan A.J., Shulruf B. (2020). Learning about dental trauma for medical students. Dent. Traumatol..

[20] Soğukpınar Önsüren A., İpek S. (2021). Are student knowledge levels and attitudes about avulsion dental trauma adequate in the faculty of medicine?. Int. Dent. Res..

[21] Farage e Rodrigues O., Landim K.L.G., Alves R.T., Machado F.C., Carrada C.F. (2021). Knowledge of avulsion of permanent teeth emergency management among undergraduate in Brazilian health care students. Pesqui. Bras. Odontopediatria Clín. Integr..

[22] Ivkošić I., Gavić L., Jerković D., Macan D., Vladislavić N.Z., Galić N., Tadin A. (2020). Knowledge and Attitudes about Dental Trauma Among the Students of the University of Split. Acta Stomatol. Croat..

[23] Ivancic Jokic N., Bakarcic D., Grzic R., Majstorovic M., Sostarek M. (2017). What general medicine students of University of Rijeka know about dental avulsion?. Eur. J. Dent. Educ..

[24] Abudan A., Baker O., Yousif A., Merchant R.C. (2022). Projected Saudi Arabian pediatric emergency consultant physician staffing needs for 2021–2030. J. Am. Coll. Emerg. Physicians Open.

[25] Al-Majed I., Murray J.J., Maguire A. (2001). Prevalence of dental trauma in 5-6- and 12-14-year-old boys in Riyadh, Saudi Arabia. Dent. Traumatol..

[26] Al-Majed I. (2011). Dental trauma among 12–15 year-old schoolgirls in Riyadh, Saudi Arabia. J. Pak. Dent. Assoc..

[27] Al-Ansari A., Nazir M. (2020). Prevalence of Dental Trauma and Receipt of Its Treatment among Male School Children in the Eastern Province of Saudi Arabia. Sci. World J..

[28] Alanzi T., Al-Habib D.K. (2020). The Use of Social Media by Healthcare Quality Personnel in Saudi Arabia. J. Environ. Public Health.

[29] Hunter L. (2012). Challenging the reported disadvantages of e-questionnaires and addressing methodological issues of online data collection. Nurse. Res..

[30] Kostopoulou E., Sinopidis X., Gidaris D., Karantaglis N., Cassimos D., Gkentzi D., Karatza A.A., Paraskakis E., Jelastopulu E., Dimitriou G. (2021). Parents under siege: The psychological impact of COVID-19 outbreak on children’s caregivers. Swiss Med. Wkly..

[31] Eysenbach G. (2004). Improving the quality of Web surveys: The Checklist for Reporting Results of Internet E-Surveys (CHERRIES). J. Med. Internet Res..

[32] Saudi Arabia—Medical Schools—Gfmer. https://www.gfmer.ch/Medical_search/Countries/Saudi_Arabia.htm.

[33] Qazi S.R., Nasir K.S. (2009). First-aid knowledge about tooth avulsion among dentists, doctors and lay people. Dent. Traumatol..

[34] Ravn J.J. (1974). Dental injuries in Copenhagen schoolchildren, school years 1967–1972. Community Dent. Oral. Epidemiol..

[35] Day P.F., Flores M.T., O’Connell A.C., Abbott P.V., Tsilingaridis G., Fouad A.F., Cohenca N., Lauridsen E., Bourguignon C., Hicks L. (2020). International Association of Dental Traumatology guidelines for the management of traumatic dental injuries: 3. Injuries in the primary dentition. Dent. Traumatol..

[36] Bourguignon C., Cohenca N., Lauridsen E., Flores M.T., O’Connell A.C., Day P.F., Tsilingaridis G., Abbott P.V., Fouad A.F., Hicks L. (2020). International Association of Dental Traumatology guidelines for the management of traumatic dental injuries: 1. Fractures and luxations. Dent. Traumatol..

[37] Fouad A.F., Abbott P.V., Tsilingaridis G., Cohenca N., Lauridsen E., Bourguignon C., O’Connell A., Flores M.T., Day P.F., Hicks L. (2020). International Association of Dental Traumatology guidelines for the management of traumatic dental injuries: 2. Avulsion of permanent teeth. Dent. Traumatol..

[38] De Brier N., Dorien O., Borra V., Singletary E.M., Zideman D.A., De Buck E. (2020). International Liaison Committee on Resuscitation First Aid Task Force. Storage of an avulsed tooth prior to replantation: A systematic review and meta-analysis. Dent Traumatol..

[39] Khattab E., Sabbagh A., Aljerian N., Binsalleeh H., Almulhim M., Alqahtani A., Alsalamah M. (2019). Emergency medicine in Saudi Arabia: A century of progress and a bright vision for the future. Int. J. Emerg. Med..

[40] Alrabiah A., Almass A.A., Al Humaidhi R.A., Alharbi N.M., Almassad G.A. (2021). Saudi medical students’ knowledge, perception, and exposure to emergency medicine. Saudi J. Emerg. Med..

[41] Yeng T., O’Sullivan A.J., Shulruf B. (2022). Medical students’ perception of an online dental trauma course in medical education. Aust. Endod. J..

[42] Aalam A., Zocchi M., Alyami K., Shalabi A., Bakhsh A., Alsufyani A., Sabbagh A., Alshahrani M., Pines J.M. (2018). Perceptions of emergency medicine residents on the quality of residency training in the United States and Saudi Arabia. World J. Emerg. Med..

[43] Zafar S., Renner M.P., Zachar J.J. (2020). Dental trauma simulation training using a novel 3D printed tooth model. Dent. Traumatol..

[44] Khatri C., Chapman S.J., Glasbey J., Kelly M., Nepogodiev D., Bhangu A., Fitzgerald J.E., Starsurg Committee (2015). Social media and internet driven study recruitment: Evaluating a new model for promoting collaborator engagement and participation. PLoS ONE.

