# Supplementary material for: Knowledge and Attitudes of Saudi Medical Students about Emergency Management of Traumatic Dental Injuries
Source: Int J Environ Res Public Health. 2022 Oct 31;19(21):14249. doi: 10.3390/ijerph192114249 (PMC9654410; doi:10.3390/ijerph192114249)
Supplement: Supplementary file 1 [file ijerph-19-14249-s001.zip › ijerph-1974091-supplementary.pdf]

Part 1: please answer the questions below

1. You belong to which Saudi institution, please write this down …………
2. What is your academic level?
   a) Fourth-year
   b) Fifth year
   c) Sixth year
3. Your institution is:
   a) Public
   b) Private
4. Did your curriculum include information about traumatic dental injuries in children and their management?
   a) Yes
   b) No
   c) do not know

Part 2: please circle the correct answer

Case I: A 9-year-old student fell down while walking, and as her face hit the pavement, she broke off the maxillary central tooth at the horizontal middle line of the crown. Otherwise, she is healthy, unhurt, and conscious.

1. The broken tooth is likely to be:
   a) Deciduous tooth
   b) Permanent tooth
   c) Do not know

2. Your immediate management of the case is:
a) Refer the patient to a dentist without advising her to keep the tooth fragment.
b) Advise the patient to save the tooth pieces or fragments and refer her to a dentist.
c) Suggest the patient to have the tooth extracted.

Case II: A 12-year-old boy was punched in the face and had a tooth knocked out. There is some blood in his mouth. Otherwise, he is healthy, unhurt, and conscious.

1. The immediate emergency action you would take is:
   a) Stop the bleeding by applying gentle pressure with a cloth over the injury and advise the patient to rest.
   b) Stop the bleeding and then search for the tooth.
   c) Look for the tooth and put it back in its socket.

d) Place the tooth in a handkerchief and refer the child to a dentist.
f) Because of the hopeless prognosis, there is no need to replant the tooth.

2. Would you investigate if the child had a tetanus vaccine?
a) Yes
b) No

3. How urgently do you think it is to replant an avulsed tooth?
a) Immediately
b) Within a few hours
c) Within the same day
d) This is not a crucial factor

4. Would you similarly care if a primary tooth is knocked out?
a) Yes
b) No

5. If the tooth has fallen on the dirty ground, what would you do?
a) Rub away the dirt with a paper tissue and put it back into its socket.
b) Clean the tooth with a toothbrush under tap water and put it back into its socket.
c) Rinse the tooth gently under tap water and put it back into its socket.
d) Discard the tooth.

6. How would you hold the tooth?
a) By the crown
b) By the root
c) Not important (crown or root)

7. Which storage medium is appropriate for storing an avulsed tooth? (you can choose more than one option)

a) Tap water	o yes o no
b) Cold water	o yes o no
c) Hot water	o yes o no
d) Salt water	o yes o no
e) Coconut water	o yes o no
f) Ice	o yes o no
g) Milk	o yes o no
h) Disinfectant solution	o yes o no
i) Patient saliva	o yes o no
j) Egg white	o yes o no
k) Coke	o yes o no
l) Normal saline	o yes o no
m) Alcohol	o yes o no
n) Contact lens solution	o yes o no
o) Plastic foils	o yes o no
p) Cell culture media	o yes o no

q) Wrap in paper tissue o yes o no

Part 3: please circle the correct answer

1: I feel confident in diagnosing different traumatic dental injuries which may affect children

a) Strongly disagree
b) Disagree
c) Neither agree nor disagree
d) Agree
e) Strongly agree

2: I can provide emergency treatment-if required- for traumatic dental injuries in children

a) Strongly disagree
b) Disagree
c) Neither agree nor disagree
d) Agree
e) Strongly agree

3: My knowledge level about traumatic dental injuries and their management in children is satisfactory

a) Strongly disagree
b) Disagree
c) Neither agree nor disagree
d) Agree
e) Strongly agree

4: I need further education about emergency management of traumatic dental injuries in children

a) Strongly disagree
b) Disagree
c) Neither agree nor disagree
d) Agree
e) Strongly agree
